# The Protective Role of *Bacillus velezensis* A2 on the Biochemical and Hepatic Toxicity of Zearalenone in Mice

**DOI:** 10.3390/toxins10110449

**Published:** 2018-10-31

**Authors:** Nan Wang, Peng Li, Mingyang Wang, Si Chen, Sheng Huang, Miao Long, Shuhua Yang, Jianbin He

**Affiliations:** Key Laboratory of Zoonosis of Liaoning Province, College of Animal Science & Veterinary Medicine, Shenyang Agricultural University, Shenyang 110866, China; 20162534@stu.syau.edu.cn (N.W.); lipeng2018@syau.edu.cn (P.L.); 20162533@stu.syau.edu.cn (M.W.); 2017220528@stu.syau.edu.cn (S.C.); 2017220530@stu.syau.edu.cn (S.H.)

**Keywords:** *Bacillus velezensis* A2, zearalenone (ZEN), feed detoxification, serum enzyme, oxidative damage

## Abstract

Zearalenone (ZEN) is an estrogen-like mycotoxin produced by *Fusarium* that seriously compromises the safety of animal and human health. In this study, our aim was to evaluate the protective effect of *Bacillus velezensis* A2 against biochemical and pathological changes induced by zearalenone in mice. Kunming mice (*n* = 40; 25 ± 2 g) were allotted to four treatment groups: a control group (basic feed); a ZEN group (basic feed with a ZEN dose of 60 mg/kg); an A2 strain fermented feed group (150 g of feed mixed with 150 mL of sterile distilled water and inoculated with 5 mL of phosphate buffer salt (PBS) resuspended A2 strain); and an A2 strain fermented ZEN-contaminated feed group. (A2 strain group 150 mL pure bacterial distilled water system mixed with 150 g ZEN-contaminated feed.) Our results showed that the *Bacillus velezensis* A2 strain can completely degrade the ZEN-contaminated feed within 5 days. (The concentration of ZEN in fermentation was 60 μg/mL.) After the mice fed for 28 days, compared with the control group, the activities of AST and ALT were increased, the activities of glutathione peroxidase (GSH-PX) and total superoxide dismutase (T-SOD) were decreased, and the amount of creatinine (CRE), blood urea nitrogen (BUN), uric acid (UA), and malondialdehyde (MDA) in the ZEN group were increased in the mice serum (*p* < 0.05; *p* < 0.01). However, compared with the ZEN group, these biochemical levels were reversed in the A2 strain fermented feed group and in the A2 strain fermented ZEN-contaminated feed group (*p* < 0.05; *p* < 0.01). Furthermore, histopathological analysis only showed pathological changes of the mice liver in the ZEN group. The results showed that *Bacillus velezensis* A2 as additive could effectively remove ZEN contamination in the feed and protect the mice against the toxic damage of ZEN. In conclusion, *Bacillus velezensis* A2 has great potential use as a microbial feed additive to detoxify the toxicity of zearalenone in production practice.

## 1. Introduction

Zearalenone (ZEN) is an estrogen-like mycotoxin that is produced by *Fusarium fungi*, and is also known as F2 toxins [1]. ZEN contamination is very serious worldwide; it is mainly found in moldy cereal crops and other cereal crop products, but it also harms animal health through the food chain [2,3,4]. Studies have shown that ZEN can cause reproductive abnormalities such as abortion, stillbirth, and deformity in female animals, which seriously endangers the reproduction of farm animals [5]. ZEN has also been shown to contain immunotoxicity [6,7,8] and cytotoxicity [9,10]. Meanwhile, ZEN can cause damage to internal organs, especially the liver [11,12]. In addition, the increase of ZEN in the food chain poses a serious hazard to human health [13,14,15].

The zearalenone residues are a serious issue for feed and food safety. Although countries have defined the ZEN maximum residue limits (MRLs) in feed and food, ZEN toxins still seriously harm the food industry and animal husbandry, since *Fusarium fungus* spores are widespread in nature. In production practice, when spores attached to cereal products are exposed to moist conditions, they rapidly grow and produce ZEN toxins. This answers why excessive mycotoxins are detected in feed and food in various countries [16,17,18].

In detoxification studies of mycotoxin, the detoxification treatment of zearalenone is usually carried out by physical, chemical, and biological methods to eliminate or attenuate the toxicity of ZEN. Nevertheless, physical and chemical methods have some limitations concerning cost of production, nutritional value, and palatability. In recent years, biological detoxification has become an important research topic for ZEN decontamination because it has the advantages of high specificity, high efficiency, and non-toxic metabolites [19]. A few studies have been done on the detoxification of ZEN by microorganisms, including yeast [20,21], *Lactobacillus* [22], *Bacillus* [23,24], *Acinetobacter* [25], *Pseudomonas* [26], *Clonostachys rosea* [27], and *Sphaerodes mycoparasitica* [28]. However, the ZEN microbial decontamination research to date has tended to focus on identifying detoxification for a specific culture medium. It has been reported that only a few microorganisms can be used to detoxify the mycotoxins in the feed and food. Therefore, the microbial detoxification strategy of mycotoxins in feed and food should be vigorously developed. Through this approach, the safety of cereal products can be improved and animal health can be protected.

Our preliminary study showed that following treatment of ZEN (7.45 μg/mL) and of the *Bacillus velezensis* A2 for 72 h in a Luria-Bertani (LB) medium, high performance liquid chromatography (HPLC) analyses revealed a ZEN-degradation efficiency of 100% [29]. In addition, another study reported that *Bacillus velezensis* can be used as a biocontrol agent for various plant diseases caused by phytopathogenic *fungi* [30,31]. Meanwhile, *Bacillus velezensis* exhibits broad spectrum antimicrobial activity against various foodborne pathogens [32,33], and there are no gene families that correlate with the human pathogenicity detected in the *Bacillus velezensis* genome [34]. In summary, *Bacillus velezensis* is a probiotic that can be used to decontaminate mycotoxins in production practice. According to the national microorganism feed additive standard (GB/T23181-2008, China), *Bacillus velezensis* conforms to the safety standard of microbial feed additive. As an additive, *Bacillus velezensis* has great potential to decontaminate mycotoxins in animal feed.

This study set out to determine the potential of *Bacillus velezensis* A2 as a feed additive for the decontamination of ZEN-contaminated feed. We evaluated the decontamination effect of the A2 strain on ZEN-contaminated feed by high performance liquid chromatography (HPLC). In addition, the growth performance, serum hormone, oxidation index, and liver pathology of Kunming mice were evaluated by managing basic feed, ZEN-contaminated feed, A2 strain fermented feed, and A2 strain fermented ZEN-contaminated feed.

## 2. Results

### 2.1. The Decontamination Effect of the A2 Strain in ZEN-Contaminated Feed

**Figure 1:**
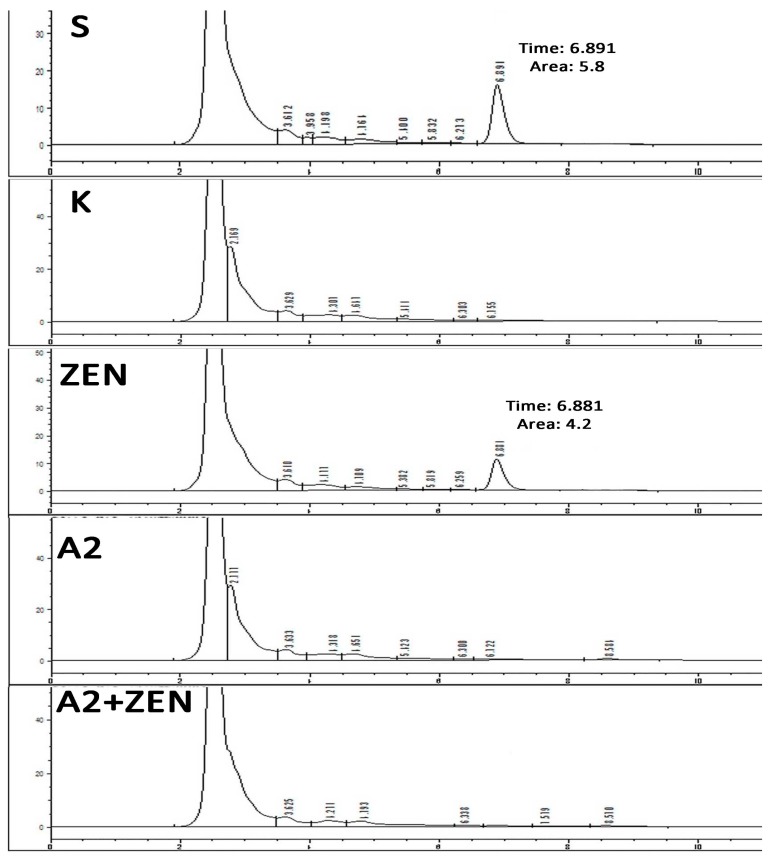
The decontamination effect of the A2 strain on Zearalenone (ZEN)-contaminated feed was determined by HPLC. (**S**) The chromatogram of the ZEN standard dissolved in methanol; the ZEN dose was 5 μg/mL, and it showed a peak at 6.891 min. (Peak area was 5.80.) (**K**) The basal feed sample was detected by HPLC after methanol extraction. (**ZEN**) The ZEN-contaminated feed sample (ZEN 60 mg/kg) was detected by HPLC after methanol extraction, and it showed a ZEN peak at 6.881 min. (Peak area was 4.20.) (**A2**) The A2 fermented feed sample was detected by HPLC after methanol extraction. (**A2 + ZEN**) The ZEN-contaminated feed sample fermented by A2 strain was detected by HPLC after methanol extraction.

Our results showed that the peak area of the ZEN standard is 5.80, which appears in 6.891 min, as shown in Figure 1. The peak area of ZEN in ZEN-contaminated feed samples was 4.20, which appeared in 6.881 min, as shown in Figure 1. Because the ZearalaTest™ column (an immunoaffinity chromatographic column, VICAM, Milford, MA, USA. Supplier: Clover Techology Group Co., Ltd. Beijing, China. Standard number: GB/T 23504-2009, China.) contains a certain amount of monoclonal antibodies, its selectively adsorbed antigens are immobilized. When the concentration of ZEN in the sample is too high, the ZEN in the sample solution cannot be completely adsorbed by the ZearalaTest™ column, resulting in a decrease in the recovery rate. The adsorption properties of the ZearalaTest™ column for different doses of ZEN were investigated. The results showed that the maximum adsorption capacity of the immunoaffinity column was about 4.0 μg. The recovery rate of ZEN was about 70.8% in the range of 0–4.0 μg of ZEN toxin. When the ZEN content was overloaded, the adsorption capacity remained basically unchanged. Therefore, according to the content of ZEN in the sample, the sample solution should be properly diluted in order to achieve the ideal recovery effect. This explains why there are lower ZEN measurements in the diet contaminated by ZEN in this experiment. In this study, the addition amount of A2 strain in A2 fermentation feed group and A2 fermentation ZEN-contaminated feed group remained the same. The feed samples contaminated by ZEN were degraded by strain A2 for 5 days. (The concentration of ZEN in fermentation was 60 μg/mL.) It maintained the same appearance and fragrance as the basic feed for A2 fermentation, so we considered that the biomass of A2 strain in A2 fermented feed group and A2 fermented ZEN-contaminated feed group was the same. As expected, no chromatographic peaks corresponding to ZEN substances were detected, which indicates that the A2 strain cleared the ZEN toxin in the feed by fermentation.

### 2.2. Changes in the Body Weight and Organ Coefficient

In the course of the experiment, the mice in each group had a normal diet, a lively posture, a neat and glossy coat, and a normal color of feces; they had no obvious symptoms of poisoning. During a 4-week test period, no death or abnormal behavior occurred in all mice. It can be seen from Table 1 that the average daily feed intake (ADFI) and average daily weight gain (ADG) of mice in the ZEN group were significantly lower than those in other groups during the period of 0–21 days. Compared with the ZEN group, the average daily intake and average daily gain of mice increased in the ZEN diet supplemented with the A2 strain. The results showed that ZEN-contaminated feed affected the diet and the weight gain of developing mice. The ZEN-contaminated feed fermented by A2 strain alleviated the adverse effects of ZEN on the developing mice. Over the next 22–28 days, the ADFI of the mice in each group tended to stabilize, and the ADG slowed down significantly. The reason for this was that the mice reached the stage of growth, development, and maturity at 22–28 days.

Table 2 shows changes in the body weight and the organ coefficient of mice in each group. At the end of each group diet trial, the organ coefficient of the liver, kidney, and testis was significantly lower in mice in the ZEN-contaminated feed group than in the basal feed group (*p* < 0.01). The organ coefficient of the spleen was significantly higher in mice in the ZEN-contaminated feed group (*p* < 0.05). However, these changes were improved in the A2 fermented ZEN-contaminated feed group compared to the ZEN-contaminated feed group. In addition, there was no significant difference in the organ coefficient between the basal feed group and the A2 fermented feed group, indicating that the A2 fermented feed did not cause harm to the health of the mice. Meanwhile, there was no significant difference between the A2 fermented ZEN-contaminated feed group and the basal feed group (*p* < 0.05). The results showed that the A2 strain could clear the ZEN in feed and prevent ZEN from impairing the health of mice.

### 2.3. Changes in Serum Biochemical Parameters

**Figure 2:**
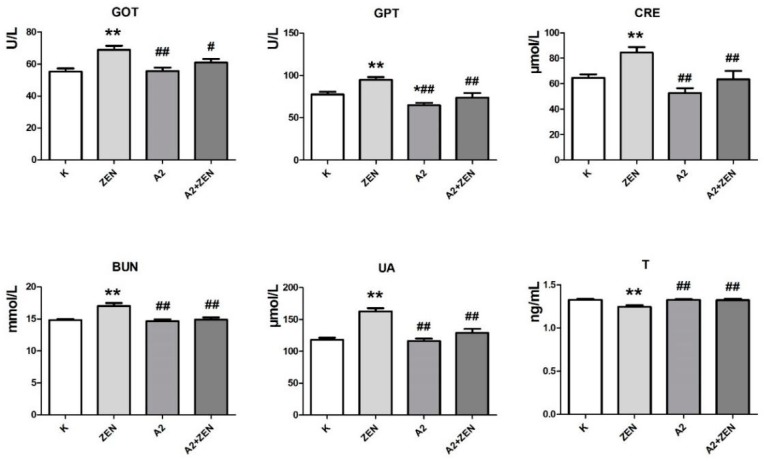
The detection results of serum biochemical parameters of mice after the 4-week diet treatment in each treatment group. The Zearalenone dose of ZEN-contaminated feed is 60 mg/kg; **K**: feeding the basal feed group; **ZEN**: feeding the ZEN-contaminated feed group; **A2**: feeding the A2 fermented feed group; **A2 + ZEN**: feeding the A2 fermented ZEN-contaminated feed group. * indicates compared to the base feed group. ^#^ indicates compared to the ZEN-contaminated feed group. “*”and “^#^” indicate significant differences (*p* < 0.05). “**” and “^##^” indicate very significant differences (*p* < 0.01). GOT: aspartate aminotransferase; GPT: alanine aminotransferase; CRE: creatinine; BUN: blood urea nitrogen; UA: uric acid; T: testosterone.

**Figure 3:**
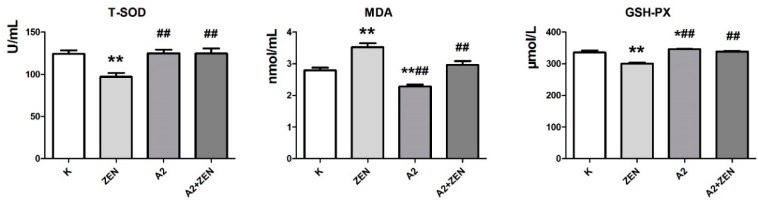
The detection results of serum oxidative damage parameters in mice after the 4-week dietary treatment in each treatment group. The Zearalenone dose of ZEN-contaminated feed is 60 mg/kg; **K**: feeding the basal feed group; **ZEN**: feeding the ZEN-contaminated feed group; **A2**: feeding the A2 fermented feed group; **A2 + ZEN**: feeding the A2 fermented ZEN-contaminated feed group. * indicates compared to the base feed group. ^#^ indicates compared to the ZEN-contaminated feed group. “*” and “^#^” indicate significant differences (*p* < 0.05). “**” and “^##^” indicate very significant differences (*p* < 0.01). T-SOD: total superoxide dismutase; MDA: malondialdehyde; GSH-PX: glutathione peroxidase.

The changes of the serum biochemical parameters in the mice of each treatment group are shown in Figure 2 and Figure 3. In the serum of all mice tested, compared with the basal feed group, the administration of ZEN-contaminated feed resulted in a significant increase in aspartate aminotransferase (GOT), alanine aminotransferase (GPT), creatinine (CRE), blood urea nitrogen (BUN), uric acid (UA), and malondialdehyde (MDA) serum levels (*p* < 0.01). Meanwhile, the administration of ZEN-contaminated feed resulted in a significant decrease in testosterone (T), total superoxide dismutase (T-SOD), and glutathione peroxidase (GSH-PX) serum levels (*p* < 0.01). These results indicate that ZEN alters the serum biochemical parameters in mice and causes oxidative damage in vivo. Similarly, feeding mice the A2 fermented ZEN-contaminated feed did not create changes in the serum biochemical parameters similar to those in the ZEN-contaminated feed group. Furthermore, in the serum of all mice tested, the A2 fermented feed group and the A2 fermented ZEN-contaminated feed group showed no different or better serum biochemical parameters compared to the basal feed group. The above results indicate that the A2 strain can remove ZEN from the feed and protect the mice from the toxic damage of ZEN.

### 2.4. Analysis of the Oxidative Parameters of Liver

**Figure 4:**
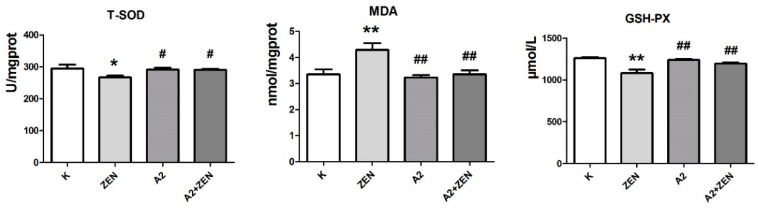
The detection results of liver oxidative damage parameters in mice after the 4-week dietary treatment in each treatment group. The zearalenone dose of ZEN-contaminated feed is 60 mg/kg; **K**: feeding the basal feed group; **ZEN**: feeding the ZEN-contaminated feed group; **A2**: feeding the A2 fermented feed group; **A2 + ZEN**: feeding the A2 fermented ZEN-contaminated feed group. * indicates compared to the base feed group. ^#^ indicates compared to the ZEN-contaminated feed group. “*” and “^#^” indicate significant differences (*p* < 0.05). “**” and “^##^” indicate very significant differences (*p* < 0.01).

MDA levels are hallmarks of lipid peroxidation, and the T-SOD and GSH-PX levels represent the body’s antioxidant capacity. As shown in Figure 4, we evaluated the liver oxidative damage in mice of each treatment group. Compared with other groups, the administration of ZEN-contaminated feed resulted in altered oxidative parameters in the liver of mice, such as a significant increase in MDA (*p* < 0.01) as well as a significant decrease in T-SOD and GSH-PX (*p* < 0.05). The results showed that the administration of ZEN-contaminated feed caused oxidative damage to the liver of mice. However, compared with the ZEN-contaminated feed group, the ZEN-contaminated feed after fermentation of the A2 strain not only reversed the ZEN-induced increase in MDA (*p* < 0.01), but also altered the ZEN-induced decrease in GSH-PX and SOD activity (*p* < 0.05). In addition, for the liver oxidation index, there were no significant differences between the three diet groups: the basal feed group, the A2 fermented feed group, and theA2 fermented ZEN-contaminated feed group. The above results indicate that the A2 strain can effectively purify the ZEN from the feed and protect the mice liver from the ZEN-induced oxidative damage.

### 2.5. Liver Histopathological Changes

**Figure 5:**
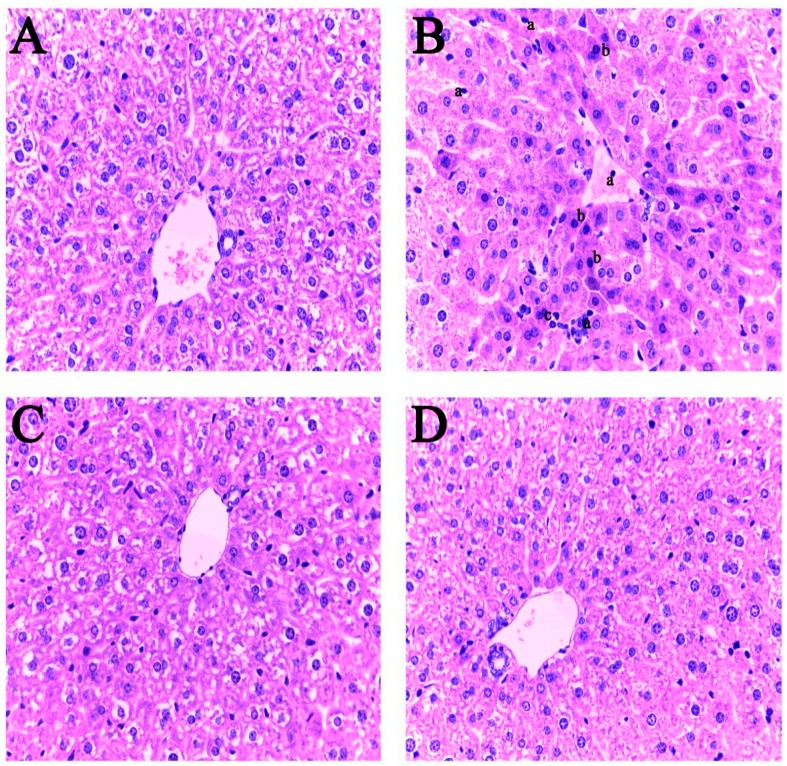
The detection results of liver histopathology of mice after the 4-week diet treatment in each treatment group (original magnification of 400×). (**A**): feeding the basal feed group; (**B**): feeding the ZEN-contaminated feed group; (**C**): feeding the A2 fermented feed group; (**D**): feeding the A2 fermented ZEN-contaminated feed group. a: lymphocytes; b: cells with nuclear atrophy and enhanced cytoplasmic staining.

In the course of dissection, no abnormality was found in the visceral organs of the mice. The naked appearance of the liver of the mice was bright red. It had a soft texture, no obvious swelling, and a smooth surface. There were no rough, bleeding, or necrosis points or other lesions. The histopathological results of liver in each treatment group are shown in Figure 5. The morphology of liver cells in the basal feed group and the A2 fermented feed group exhibited normal cells with a clear cytoplasm and an intact nucleus, as shown in Figure 5A,C. However, in contrast, the ZEN-contaminated feed revealed pathological changes in liver tissue; these pathological changes focused on nuclear atrophy, enhanced cytoplasmic staining, and lymphocytic infiltration, as shown in Figure 5B. However, no pathological changes in the liver were found in the A2 fermented ZEN-contaminated feed group, as shown in Figure 5D. These results indicate that the A2 strain can decontaminate ZEN in feed and protect mice liver from the ZEN-induced toxic damage.

## 3. Discussion

According to previous research, *Bacillus velezensis* A2 acts as a microbial antagonist, with a highly effective and stable ZEN-decontamination capability, and it has a protective effect on kidney injury induced by ZEN in mice [29]. In addition, recent studies have shown that *Bacillus velezensis* DY 3108 has high degradation ability to aflatoxin B1 (AFB 1) [35]. This shows that *Bacillus velezensis* has a broad application prospect in mycotoxin detoxification and feed substrate bioremediation. In this study, we evaluated the potential value of *Bacillus velezensis* A2 as a feed additive; in vitro experiments showed that the fermentation of *Bacillus velezensis* A2 could completely remove ZEN from the feed and that the ZEN-contaminated feed after A2 strain fermentation would not cause damage to the Kunming mice. This discovery will greatly facilitate the practice of microbial decontamination of mycotoxins in feeds.

Many microorganisms capable of degrading mycotoxins have been identified in the field of detoxification of mycotoxins. For instance, it has been confirmed that in the process of mycotoxin decontamination, the microbial cell wall has a good adsorption effect on ZEN (such as yeast [36], *Lactobacillus plantarum* [22]), and the microbial secretase has an efficient degradation effect on ZEN (such as *Bacillus subtilis* [24], *Bacillus licheniformis* [37], *Bacillus amyloliquefaciens* [38], and *Rhodococcus pyridinivorans K408* [39]). In the pre-test of the ZEN detoxification study, we isolated and identified a new bacterial strain (*Bacillus velezensis* A2) for the ZEN decontamination strategy [29]. Similarly, in this study, our results also showed that ZEN (60 mg/kg) was completely removed from ZEN-contaminated feed after 5 days of fermentation with the A2 strain. 

However, at the point of ZEN-degrading bacteria currently found, the metabolic process of most microorganisms against ZEN cannot be considered as detoxification. Because the components of these microbial metabolic pathways are unclear or maintain estrogen-like effects similar to ZEN, this limits their detoxification of mycotoxins in food and feed production practices. For example, α-ZOL and β-ZOL produced during microbial transformation still have estrogen-like toxicity [40]. The masked mycotoxins (ZEN-*O*-14-glucoside, ZEN-*O*-16-glucoside, ZEN-14-sulfate) were produced during the microbial transformation, and they were easily cleaved by digestive enzymes or microorganisms in the gastrointestinal tract of animals, releasing ZEN [41,42]. Therefore, it is necessary to establish a combined in vitro and in vivo detection strategy to assess whether the A2 strain is feasible in the practical application of mycotoxin detoxification. Although the mechanism of detoxification of ZEN by the *Bacillus velezensis* A2 strain is not clear, the safety evaluation test of mice confirmed that the A2 strain as a microbial additive has great potential for the detoxification of mycotoxins in feed. In subsequent studies, we will further uncover the mechanism of ZEN detoxification by *Bacillus velezensis* A2.

In the feeding trial, we evaluated whether the fermentation of ZEN-contaminated feed by *Bacillus velezensis* A2 could improve the adverse effects of ZEN on Kunming mice. Our findings suggest that the growth performance and serum biochemical parameters of mice changed after managing the ZEN-contaminated feed. This is consistent with previous reports indicating that the ZEN diet resulted in a significant increase in GOT, GPT, CRE, BUN, UA, and MDA serum levels in mice (*p* < 0.01) [43,44,45,46]. In addition, ZEN caused the decrease of the liver, kidney, and testis coefficients and the increase of the spleen coefficient, which seriously affected the growth and development of the Kunming mice. The liver is an important target organ of estrogen receptors, and the ZEN-induced liver oxidative damage in mice has been reported many times [47]. In the course of dissection, no abnormality was found in the visceral organs of the mice. The appearance of the liver in the mice was bright red, with a soft texture, no obvious swelling, and a smooth surface. There were no rough, bleeding, or necrosis points or other lesions. To further understand the damage of ZEN to mice, this study also investigated the oxidative stress and pathological changes in the liver. In our study, the administration of the ZEN-contaminated feed showed similar changes to the oxidation parameters in the liver of mice [47], such as a significant increase in MDA (*p* < 0.01) as well as a significant decrease in T-SOD and GSH-PX (*p* < 0.05).

In a few cases, microbial additives have been reported for use in mycotoxin decontamination feeding strategies. Studies have shown that the addition of yeast products to basal diets can reduce the effects of mycotoxins on pig growth and health [48]. The fermentation of *Bacillus licheniformis* CK1 could effectively degrade ZEN in feed, and reduce the adverse effects of ZEN on pigs [49]. In this study, under the same management conditions, the mice in the A2 fermented feed group had a similar growth index (ADFI, ADG, and organ coefficient), serum biochemical index, and oxidation parameters to those in the basal feed group. Compared with the ZEN-contaminated feed group, changes to the liver, kidney, testis, and spleen coefficients induced by ZEN were not found in the A2 fermented ZEN-contaminated feed group. It means that *Bacillus velezensis* A2 can remove ZEN from feed and protect mice from the toxic damage of ZEN. Furthermore, a similar biological additive detoxification test has also been reported in mice. For example, when *Lactobacillus rhamnosus* GG (LGG) was added to the feed, it improved the intestinal barrier function and treated the DON/ZEA-induced adverse reactions in mice [50].

## 4. Conclusions

The results showed that using *Bacillus velezensis* A2 as an additive could effectively remove ZEN contamination in feed and protect the mice against the toxic damage of ZEN. Although the mechanism of detoxification of ZEN by the *Bacillus velezensis* A2 strain is not clear, the safety evaluation test of mice confirmed that the A2 strain as a microbial additive has great potential for the detoxification of mycotoxins in feed.

## 5. Materials and Methods

### 5.1. Chemicals, Strains, and Culture Conditions

The zearalenone (ZEN) standard and the chromatographic grade methanol were purchased from Sigma (St. Louis, MO, USA).

Shenyang Agricultural University isolated the *Bacillus velezensis* A2, and this strain was deposited with the China Center for Type Culture Collection (CCTCC: M2018352).

The Luria-Bertani (LB) stock solution (500 mL: 5 g NaCl, 2.5 g yeast extract, and 5 g tryptone) was adjusted to pH 7.2 using 4 M NaOH. It was autoclaved at 121 °C for 20 min. (The culture medium was sterilized and the singularity of the tested strains was ensured in the study.) For production of a solid medium, 7.5 g/L of agar powder was added.

The A2 strain was streaked on an LB Agar plate of PH = 7.2 and incubated in bacteriological incubator for 48 h at 37 °C. Single colonies were inoculated into a 30 mL LB medium (autoclave sterilization of 30 mL PH = 7.2 LB medium in 50 mL conical bottle), and then transferred to a constant temperature oscillator to incubate in a fixed orbit shaking bed for 48 h at 37 °C, 150 rpm to prepare A2 bacteria solution.

### 5.2. Detection of ZearalaTest™ Column Recovery Rate

**Figure 6:**
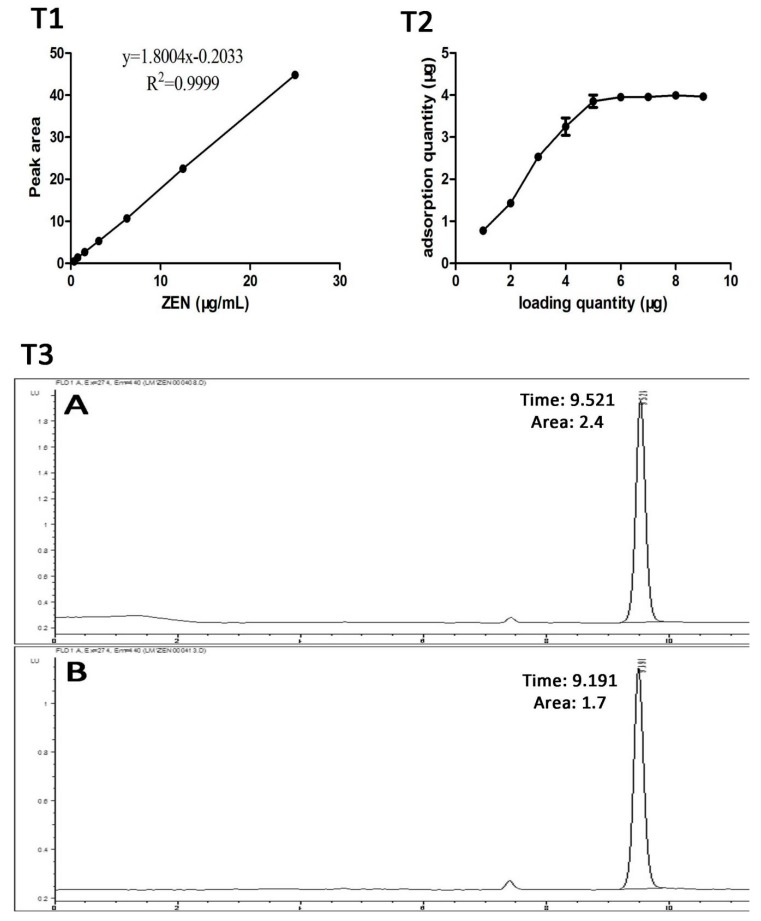
The determination of the recovery rate of the ZearalaTest™ column and the determination of the standard curve for the ZEN toxin. (**T1**): the ZEN standard curve. Under chromatographic conditions, linear regression analysis was carried out with the standard concentration (X) as the horizontal coordinate and the peak area (Y) as the vertical coordinate. (**T2**): adsorption curve of the ZearalaTest™ column immunoaffinity column. (**T3**): Recovery rate of ZearalaTest™ column immunoaffinity column. (A: The ZEN standard solution with 1 mL concentration of 1.5 μg/mL was used for direct determination. The chromatographic peak appeared at 9.521 min, and the peak area was 2.4. B: The standard solution of ZEN with 1 mL concentration of 1.5 μg/mL was recovered by the ZearalaTest™ column, and the recovered ZEN was dissolved in 1 mL methanol for HPLC detection. The chromatographic peak appeared at 9.191 min, and the peak area was 1.7.) The peak area was brought into the linear regression equation to calculate the sample content. Percent recovery (%) = adsorbing quantity/loading quantity.

The ZEN standard material 5.00 mg was extracted accurately and dissolved in 2 mL chromatographic methanol to prepare the ZEN reserve solution. The ZEN reserve solution was diluted with 50% methanol water (volume fraction) to 25, 12.5, 6.25, 3.125, 1.5625, 0.78125, and 0.390625 μg/mL. Under chromatographic conditions, linear regression analysis was carried out with the standard concentration (X) as the horizontal coordinate and the peak area (Y) as the vertical coordinate. The linear regression equation is as Equation (1). In the range of 0.39–25.00 μg/mL of the ZEN dose, the peak area of ZEN is linearly related to the concentration of ZEN, as shown in Figure 6T1. Therefore, in subsequent tests, the LOD and LOQ for the C18 column were set to 0.39 ppm and 25 ppm, respectively.

y = 1.8004x − 0.2033, R^2^ = 0.9999 (1)

Because the ZearalaTest™ column (VICAM, Milford, MA, USA. Supplier: Clover Techology Group Co., Ltd., Beijing, China. Standard number: GB/T 23504-2009, China) contains a certain amount of monoclonal antibodies, its selectively adsorbed antigen is immobilized. When the content of ZEN in the sample solution is too large, the ZEN in the sample solution cannot be completely adsorbed by the immunoaffinity column when it passes through the ZearalaTest™ immunoaffinity column, resulting in a decrease in the recovery rate [18]. In this study, 1–10 μg ZEN dissolved in 2 mL methanol was used to evaluate the recovery of ZearalaTest™ column. The results showed that the recovery was about 70.8% when the ZEN quality was less than 4.0 μg, as shown in Figure 6(T2,T3). When the content of ZEN is too high, the highest recovery is about 4.0 μg. Therefore, according to the content of ZEN in the sample, the sample solution should be diluted properly in order to achieve the ideal recovery effect.

The specific conditions of high performance liquid chromatography (HPLC) detection were as follows: Agilent ZORBAX SB-C18 (4.6 mm × 250 mm, 5 μm); sample size: 20 μL; mobile phase: methanol/water (80/20); flow rate: 1.0 mL/min; fluorescence detector; excitation wavelength is 274 nm and emission wavelength is 440 nm. The percentage of ZEN removal was calculated using the following formula: 1 − (peak area of ZEN in the supernatant/peak area of ZEN in the positive control) × 100%. 

### 5.3. The Determination of ZEN Residues in Feed by High Performance Liquid Chromatography (HPLC)

Previous studies have reported that the administration of ZEN (10–30 mg/kg BW) for 4 weeks resulted in serum hormone changes and organ damage in mice [43,51,52]. In this study, all mice were free to eat and drink. The daily feed intake of the mice was not fixed, but the average feed intake of the mice during the adaptation was 6.0 g/day. Therefore, the ZEN 60 mg/kg dose was chosen to be added to the feed in order to ensure that the daily intake of ZEN in mice was limited to the range of 15 ± 2 mg/kg BW, since the dosage of ZEN 60 mg/kg is not common in the natural occurrence of fodder mycotoxins. To ensure the normal decontamination test of feed toxin, 150 g feed was added to the disinfectant distilled water of 150 mL mixed A2 strain (Bacteria were collected from 5 mL A2 solution by centrifugation of 8000 rpm for 5 min.). The concentration of the ZEN toxin during A2 fermentation was 60 μg/mL. The programs are as follows.

Specific pathogen free (SPF) grade small mice maintenance fodder was purchased from Changchun Biotechnology of China. (For feed ingredients, ZEN ≤ 0.2 ppm, the concentration was too low and not in our range; see Supplementary Table S1) The feed was pulverized by a pulverizer and divided into four groups, and 150 g/group of the powder was placed in a 250 mL conical flask for autoclaving. The basal feed group: 150 g of feed powder was mixed with 150 mL of sterile distilled water; the ZEN feed group: ZEN (9 mg) was dissolved in 50 mL of ethanol and mixed with 150 g of feed powder. After drying at 56 °C, it was mixed with 150 mL of sterilized distilled water; the A2 fermented feed group: the pure bacterial strain from the 5 mL centrifuged A2 bacterial solution was isolated, and it was added to 150 mL of sterile distilled water, then mixed with 150 g of feed powder; the A2 fermented ZEN feed group: the A2 strain group with a 150 mL pure bacterial distilled water system was mixed with 150 g of ZEN-contaminated feed. All treatment group samples were incubated at 37 °C for 5 days in an orbital shaker at 150 rpm. The fermented feed powder was poured into a stainless steel tray, and placed in a drying oven at 60 °C until it could be kneaded. After pelletizing with a granulator, it was completely dried at 60 °C.

The samples were analyzed according to the certified Chinese GB/T 23504-2009 method [18]. In this study, 12.50 g of the feed samples (accurate to 0.01 g) were extracted from each group, then crushed and put into 250 mL beaker. After adding 2.5 g NaCl, 100 mL 80% methanol water (volume fraction) solution to the magnetic agitator, it was used to agitate the solution for 5 min at a high speed. Filtered with qualitative filter paper, collected filtrate into 50 mL centrifuge tube, centrifugation of 5000 r/min for 5 min. Then, transferred the upper liquid 10 mL to the new centrifuge tube and added 40 mL phosphate buffer salt (PBS, 0.01 M, PH = 7.2) solution. The solution was mixed well and centrifuged for 5 min at 10,000 r/min. The supernatant of 10 mL was collected, and the supernatant was passed through the ZearalaTest™ column at the flow rate of 1–2 drops/s (ZEN reserve solution was diluted to 0.5 μg/mL with PBS, take 10 mL for the same treatment). It was then dripped washed twice with water (10 mL each time) until air entered the affinity column. All the outflow of liquid from the pump dry column was then discarded. The bound ZEN in column was eluted by 2.0 mL methanol at a flow rate of 1.0 mL/min. All eluent was collected in a glass tube and dried with nitrogen below 55 °C. The residue was dissolved in 1 mL 50% methanol water and filtered with a 0.22 μm microporous membrane. The diluent was filtered by a 0.22 μm microporous membrane, and then a 20 μL filtrate was injected into high performance liquid chromatography (HPLC) system to analyze the content of ZEN. For test conditions, see Section 5.2 and Section 5.3. (There were more than 3 repetitions per group.)

### 5.4. Animals

Male Kunming mice (25 ± 2 g and 4 weeks old) were purchased from Liaoning Changsheng Biotechnology of China. Kunming male mice were selected because ZEN is an estrogenic mycotoxin that also harms the reproductive organs of male mice. Male mice can allow the observation of the effects of ZEN on testis development (testis atrophy and other abnormalities). The mice were maintained under SPF conditions with restricted access with the humidity of 45–55%; they were maintained in 12 h light/dark cycles at a temperature of 23 ± 2 °C. Before the experiment began, the mice were given an acclimatization period of 1 week. These experiments were performed in accordance with the European Communities Council Directive of 24 November 1986 (86/609/EEC) and the principles of SPF laboratory animal care. The experimental procedures have been approved by the Ethics Committee for Laboratory Animal Care (Animal Ethics Procedures and Guidelines of the People’s Republic of China) for the use of Shenyang Agricultural University, China (Permit No. 264 SYXK<Liao>2011-0001, 20, October, 2011).

### 5.5. Experimental Design and Treatment

Forty mice were randomly divided into 4 groups (basic diet group; ZEN diet group; A2 fermented diet group; A2 fermented ZEN diet group), and the average body weight of each group was recorded. During a 4-week test period, each group of diets was prepared at the same time and stored in resealable bags before feeding; animals had free access to homemade feed (see Section 5.4) and water. The mental state, behavior, diet, weight change, and death of the mice in each group were observed and recorded every day. At the end of the experiment, the average body weight of each group of mice was recorded. Blood samples were collected and were centrifuged at 7500 rpm for 15 min in a 4 °C centrifuge; serum was collected and stored at −20 °C. The mice were killed in a humane manner, and the liver, kidney, spleen, and testis were isolated and weighed. The organ coefficient was then calculated. (Organ coefficients were calculated as the organ wet weight percentage of the total body weight.) All livers were cut into two equal sections; one was placed in a tissue fixative and one was placed in a cryogenic vial and stored at −80 °C until further use.

### 5.6. Serum and Liver Tissue Biochemical Analysis

The average blood collection of mice was 1.2 mL, and about 300 μL of serum was isolated. The appearance of serum was clear and transparent; only a few of the serum in the ZEN group showed hemolysis. Serum samples were analyzed for aspartate aminotransferase (GOT), alanine aminotransferase (GPT), creatinine (CRE), blood urea nitrogen (BUN), uric acid (UA), malondialdehyde (MDA), glutathione peroxidase (GSH-PX), and total superoxide dismutase (T-SOD), using commercial kits obtained from Nanjing Jiancheng Biological Engineering (Nanjing, China). The serum testosterone was determined by the Elabscience testosterone (T) kit (Elabscience Biotechnology Co., Ltd., Wuhan, China). Meanwhile, the liver function was also assessed by analyzing the T-SOD, MDA, and GSH-PX levels of 10% liver tissue homogenate. Each measurement was determined according to the instructions of the commercial kit.

### 5.7. Liver Biochemical Assays

The liver in the tissue fixative was taken out, embedded in paraffin, and subjected to liver sectioning. The liver sections were then subjected to HE staining and examined using a microscope (Slices were produced and analyzed by China Seville Biotechnology Co., Ltd., Wuhan, China).

### 5.8. Statistical Analysis

The SPSS 19.0 software (IBM, Armonk, NY, USA) was used to carry out all statistical tests, and the results are presented as mean ± standard error (X ± SE). Multigroup comparisons of the means were carried out by one-way analysis of variance (ANOVA) test with post hoc contrasts by Student–Newman–Keuls test. The statistical significance for all tests was set at *p* < 0.05.

## Figures and Tables

**Table 1:** Effects of zearalenone and its degrading strains on the growth performance of mice.

Items		Group			
		K	ZEN	A2	A2 + ZEN
ADFI (g)	0–7 d	5.92 ± 0.57	4.92 ± 0.41 **	5.87 ± 0.32 ^##^	5.46 ± 0.33 ^#^
	8–14 d	6.18 ± 0.23	5.59 ± 0.52 *	5.97 ± 0.28 ^#^	6.00 ± 0.18 ^#^
	15–21 d	6.09 ± 0.33	5.42 ± 0.25 **	5.94 ± 0.45 ^##^	5.81 ± 0.27 ^#^
	22–28 d	5.99 ± 0.46	5.63 ± 0.28	5.96 ± 0.24	5.92 ± 0.45
ADG (g)	0–7 d	1.66 ± 0.35	1.24 ± 0.07 *	1.56 ± 0.09 ^#^	1.59 ± 0.16 ^#^
	8–14 d	1.09 ± 0.11	0.85 ± 0.09 **	0.99 ± 0.13 ^#^	0.96 ± 0.08
	15–21 d	0.74 ± 0.02	0.65 ± 0.03 **	0.69 ± 0.04	0.68 ± 0.05 *
	22–28 d	0.62 ± 0.02	0.59 ± 0.04	0.60 ± 0.03	0.58 ± 0.03

Data shown are mean ± SD (standard deviation, *n* = 10); ADFI: average daily feed intake, ADG: average daily weight gain; the zearalenone dose of ZEN-contaminated feed is 60 mg/kg. **K**: feeding the basal feed group; **ZEN**: feeding the ZEN-contaminated feed group; **A2**: feeding the A2 fermented feed group; **A2 + ZEN**: feeding the A2 fermented ZEN-contaminated feed group. In the same row: * indicates compared to the base feed group; # indicates compared to the ZEN-contaminated feed group; “*”and “^#^” indicate significant differences (*p* < 0.05); “**” and “^##^” indicate very significant differences (*p* < 0.01).

**Table 2:** The body weight and the organ coefficient of mice in different treatment groups.

Items	Group			
	K	ZEN	A2	A2 + ZEN
Weight (g)	44.79 ± 1.89	44.39 ± 1.73	45.51 ± 1.85	44.59 ± 2.70
Liver coefficient	0.0459 ± 0.0052	0.0434 ± 0.0023 *	0.0453 ± 0.0027	0.0458 ± 0.0014
Kidney coefficient	0.0160 ± 0.0007	0.0141 ± 0.0013 **	0.0159 ± 0.0005 ^##^	0.0156 ± 0.0005 ^##^
Spleen coefficient	0.0025 ± 0.0002	0.0031 ± 0.0007 *	0.0024 ± 0.0005 ^#^	0.0028 ± 0.0005
Testis coefficient	0.0073 ± 0.0004	0.0061 ± 0.0007 **	0.0074 ±0.0004 ^##^	0.0073 ± 0.0006 ^##^

Data shown are mean ± SD (standard deviation, *n* = 10); the zearalenone dose of ZEN-contaminated feed is 60 mg/kg; the organ coefficient = organ weight/body weight; **K**: feeding the basal feed group; **ZEN**: feeding the ZEN-contaminated feed group; **A2**: feeding the A2 fermented feed group; **A2 + ZEN**: feeding the A2 fermented ZEN-contaminated feed group. In the same row: * indicates compared to the base feed group; # indicates compared to the ZEN-contaminated feed group; “*” and “^#^” indicate significant differences (*p* < 0.05); “**” and “^##^” indicate very significant differences (*p* < 0.01).

## Supplementary Materials

The following are available online at http://www.mdpi.com/2072-6651/10/11/449/s1, Table S1: Main ingredients of specific pathogen free (SPF) grade small mice maintenance fodder.
