# Supplementary material for: The Protective Role of Bacillus velezensis A2 on the Biochemical and Hepatic Toxicity of Zearalenone in Mice
Source: Toxins (Basel). 2018 Oct 31;10(11):449. doi: 10.3390/toxins10110449 (PMC6267044; doi:10.3390/toxins10110449)
Supplement: Supplementary file 1 [file toxins-10-00449-s001.pdf]

toxins

MDPI

Supplementary Materials: The Protective Role of *Bacillus velezensis* A2 on the Biochemical and Hepatic Toxicity of Zearalenone in Mice

Nan Wang, Peng Li, Mingyang Wang, Si Chen, Sheng Huang, Miao Long, Shuhua Yang and Jianbin He

Table S1. Main ingredients of specific pathogen free (SPF) grade small mice maintenance fodder.

**Product Composition Analysis Guaranteed Value (per kg of feed)**							
**Product Name**	**Crude Protein**	**Crude Fat**	**Crude Fiber**	**Coarse Gray**	**Ca**	**P**	**Ca:P**
**SPF Grade Large and Small Mice Keep Feed**	≥ 180 g	≥ 40 g	≤ 50 g	≤ 80 g	10–18 g	6–12 g	1.2:1–1.7:1

**Main Nutrients of the Product (per kg of feed)**							
Fe	≥ 100 mg	V_A	≥ 7000 IU	V_{B1}	≥ 8 mg	Lys	≥ 8.2 g
Mn	≥ 75 mg	V_D	≥ 800 IU	V_{B2}	≥ 10 mg	Met + Cys	≥ 5.3 g
Cu	≥ 10 mg	V_E	≥ 60 IU	V_{B6}	≥ 6 mg	Arg	≥ 9.9 g
Zn	≥ 30 mg	V_K	≥ 3 mg	V_{B12}	≥ 0.02 mg	Histidine	≥ 4 g

**Product Chemical Pollutant Index**					
**Items**	**Index**	**Items**	**Index**	**Items**	**Index**
As, mg/kg	≤ 0.7	$C_6H_6Cl_6$, mg/kg	≤ 0.3	Cd, mg/kg	≤ 0.2
Pb, mg/kg	≤ 1.0	DDT, mg/kg	≤ 0.2	Hg, mg/kg	≤ 0.02
AFB1, ppm	≤ 0.2	ZEN, ppm	≤ 0.2		

Raw material composition: corn, soybean meal, flour, bran, fish meal, salt, calcium bicarbonate, stone powder, multivitamins, various trace elements, amino acids, etc.
